# A Multivocal Literature Review on Growing Social Engineering Based Cyber-Attacks/Threats During the COVID-19 Pandemic: Challenges and Prospective Solutions

**DOI:** 10.1109/ACCESS.2020.3048839

**Published:** 2021-01-01

**Authors:** Mohammad Hijji, Gulzar Alam

**Affiliations:** 1 Computer Science DepartmentUniversity of Tabuk Tabuk 47512 Saudi Arabia; 2 Information and Computer Science DepartmentKing Fahd University of Petroleum and Minerals 48080 Dhahran 31261 Saudi Arabia

**Keywords:** Multivocal literature review, social engineering, COVID-19, security and privacy, prospective solutions, cyber-attacks and threats

## Abstract

The novel coronavirus (COVID-19) pandemic has caused a considerable and long-lasting social and economic impact on the world. Along with other potential challenges across different domains, it has brought numerous cybersecurity challenges that must be tackled timely to protect victims and critical infrastructure. Social engineering–based cyber-attacks/threats are one of the major methods for creating turmoil, especially by targeting critical infrastructure, such as hospitals and healthcare services. Social engineering–based cyber-attacks are based on the use of psychological and systematic techniques to manipulate the target. The objective of this research study is to explore the state-of-the-art and state-of-the-practice social engineering–based techniques, attack methods, and platforms used for conducting such cybersecurity attacks and threats. We undertake a systematically directed Multivocal Literature Review (MLR) related to the recent upsurge in social engineering–based cyber-attacks/threats since the emergence of the COVID-19 pandemic. A total of 52 primary studies were selected from both formal and grey literature based on the established quality assessment criteria. As an outcome of this research study; we discovered that the major social engineering–based techniques used during the COVID-19 pandemic are phishing, scamming, spamming, smishing, and vishing, in combination with the most used socio-technical method: fake emails, websites, and mobile apps used as weapon platforms for conducting successful cyber-attacks. Three types of malicious software were frequently used for system and resource exploitation are; ransomware, trojans, and bots. We also emphasized the economic impact of cyber-attacks performed on different organizations and critical infrastructure in which hospitals and healthcare were on the top targeted infrastructures during the COVID-19 pandemic. Lastly, we identified the open challenges, general recommendations, and prospective solutions for future work from the researcher and practitioner communities by using the latest technology, such as artificial intelligence, blockchain, and big data analytics.

## I. Introduction

Social engineering (SE) is a method frequently used by hackers and cybercriminals for building strategies to trick people into granting them access to a system by breaking security best practices and standards illegally or even without breaking the law. SE tactics are used for a wide variety of malicious events enabled through human interactions. More explicitly, humans are the weakest links in cybersecurity [1]–[4]. SE attempts typically achieve success through one or more steps depending on the ability of the attackers to exploit the victim using psychological manipulations to trick users into making security mistakes, granting them access to sensitive information. The social engineer performs their role as a fraudster, and making an effort to get access to computer networks, sensitive data, and information [2]. Major social engineering cyber-attacks are accomplished through social media platforms such as Facebook, Twitter, Instagram, Snapchat, and YouTube [2], [4], [5].

In the current situation of the novel coronavirus (COVID-19) pandemic, social engineering is one of the most significant security threats faced by different organizations in both the public and private sectors, as well as end-users [2], [4], [6]. According to a CyberEdge [7] report, “the number of organizations hit with at least one successful social engineering attack per year is around 79%.” Similarly, 99% of cyberthreats were observed and executed through human interactions and done with the assistance of social engineering approach [8]. COVID-19, also known as coronavirus pandemic, is a viral disease that first identified in December 2019 in Wuhan, China caused by the “severe acute respiratory syndrome coronavirus 2 (SARS-CoV-2; formerly called 2019-nCoV)” [9]. COVID-19 spread with a rapid speed around the world, infecting millions of people in over 188 countries with a high death rate compared to other diseases, reaching over a million fatalities so far [10]. Perc *et al.* [105] proposed a method to determine the daily growth rates to reduce the risk of global spread of the COVID-19 pandemic. Similarly, Hâncean *et al.* [106] proposed human-to-human transmission networks and dispersion mechanism of the novel coronavirus. Hâncean *et al.* [106] shown the spread of novel coronavirus and they inspected the number of cases and deaths during the COVID-19 of the Brazilian cities’ populations.

National and international association are essential for combating COVID-19 and other probable epidemics to be more organized for pandemics as early as possible [107]. Science and technology play a significant role in combating COVID-19. Technology assists the research and development by producing drugs, researching vaccines, and providing testing toolkits to overcome this severe pandemic using the emerging technologies such as artificial intelligence, 5G networking, cybersecurity, blockchain, and big data [11].

The motivation behind our research work is that there is no Multivocal Literature Review (MLR) relevant to the rise of social engineering–based cyber-attacks/threats during the COVID-19 pandemic. Also, to identify the main challenges and proposed prospective solutions for social engineering–based cyber-attacks/threats. We systematically conducted this review by following the well-known published standard guidelines [22] and carefully reviewed both formal and grey literature studies. This review will help different organizations and online working-based employees to carry on their work in a secure manner. The main objective of our research study is to detect the state-of-the-art and state-of-the-practice social engineering–based techniques, attack methods, and platforms used for conducting cyber-attacks/threats with economic and societal impacts on various organizations. Similarly, we aim to identify the most targeted critical infrastructures and organizations that are exploited by cybercriminals during the COVID-19 pandemic. This research work provides an MLR related to the rise of social engineering–based cyber-attacks/threats during the COVID-19 pandemic from its start until October 2020.

The proposed MLR study is structured as follows. Section 2 comprehensively explains the social engineering definitions, types, approach, and goals involved. The detailed research methodology is discussed in section 3. Sections 4 and 5 explain the results and discussions from the conducted MLR study. Section 6 explores the motivation behind social engineering cyber-attacks and threats. Finally, section 7 provides the limitations of the study and section 8 presents the conclusion and potential future work from the completed research study.

## II. Social Engineering: Definition, Approach and Goals

**FIGURE 1.**
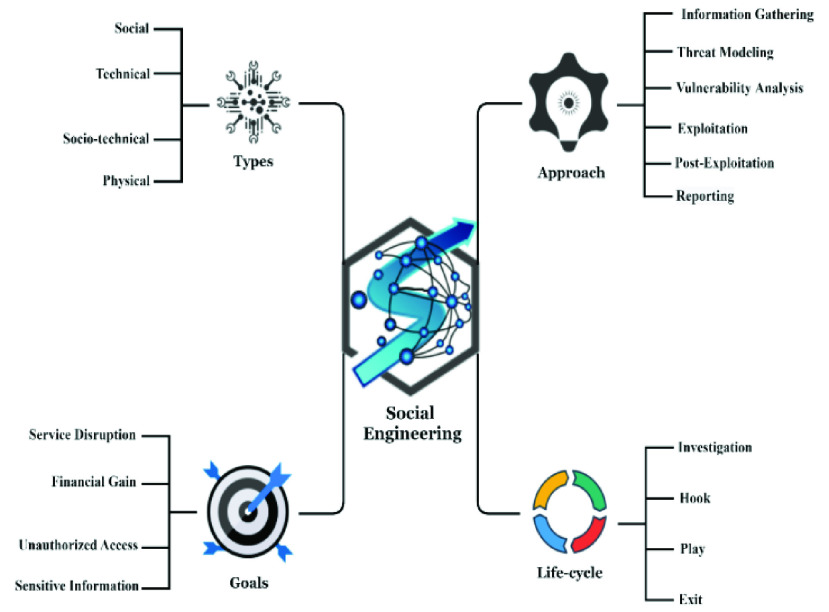
Abstract view of the social engineering goals, types, approach and lifecycle.

Social engineering “is the ultimate con—the bag of tricks employed by fraudsters who lie, cheat and steal their way past your organization’s security controls. Their goals: theft, fraud or espionage [12].” Social engineering circumvents all technologies, as well as firewalls. It appeals to hackers because people’s lack of awareness often makes their efforts easier. The comprehensive structure of social engineering is shown in Figure 1, including its primary types, approaches, life cycle, and goals [1], [13].

### A. Types

According to Krombholz *et al.* [2] and Koyun and Janabi [14], social engineering is mainly divided into four types as discussed below.

#### 1) Physical

In this type of social engineering approach, the attackers perform some actions like searching for personal data, manuals, memos, and sensitive information in trash and dumpsters. The primary purpose of the attacker is to accumulate information about the victim from physical materials.

#### 2) Social

This is the most widely used type of social engineering, in which the social engineers use psychological techniques to convince the target user with tactics like building a relationship, spear phishing, baiting, and reverse social engineering. The most commonly used social techniques for cyber-attacks are phishing, smishing, and vishing conducted via emails, texts, and phone calls.

#### 3) Technical

The technical type is usually carried out over the internet, where social networking sites are esteemed sources of information. Social engineers frequently use search engines to collect relevant information about the victims. The hackers guess or attempt to crack passwords to collect critical information about the target user. Correspondingly, the hackers and cybercriminals use automated tools as well, such as Matego and Social-Engineer Toolkit (SET) for successful cyber-attacks.

#### 4) Socio-Technical

Socio-technical techniques are the most powerful of social engineering, combining both the social and technical types. The social engineer considers certain factors like social culture of the victim, human behavior, technologies used, and building infrastructure, as well as goals and values [15]. The combination of both social and technical methods heightens the chances of successful social engineering cyber-attacks.

### B. Approach

#### 1) Information Gathering

Information gathering is the most significant phase for social engineers, where they collect and combine every piece of relevant information about the victim. It is the most exhausting and time-consuming part of the attack approach in social engineering. Most social engineers use automated online tools for information gathering by accessing the location, mobile number, and address of the target victim. Attackers apply different methods for getting organizational and individual information, such as soft skills and technical skills, depending the targets. Dumpster diving is one general way of gathering information, including medical records, emails, personal photos, bank statements, resumes, account details, tech support logs, software details, websites visited, and social media handles [16].

#### 2) Threat Modeling

Threat modeling is a procedural process for discovering the weak points in a system’s security. Social engineers try to find bugs or weaknesses in the system to take advantage of while attempting cyber-attacks. A threat model must include the current status of the system and its security, the possibility of new threats, and finally, a mitigation strategy for when the attackers deploy cyber threats. Most importantly, the threat modeling necessitates a rich understanding of objectives and the assets to be protected, along with other environmental factors [17].

#### 3) Vulnerability Analysis

Social engineers use a collection of strategies to exploit the vulnerabilities of an organization and individuals to take advantage of the system and gain access to sensitive information. Vulnerability analysis consists of four main steps: an initial assessment of the victim’s personality, behaviors, a diagnosis of vulnerabilities in the system, a selection of relevant strategies for successful exploitation of the resources, and vulnerability detection. Then the attackers develop a personalized tactics for cyber-attacks [18]. Attackers often use vulnerability scanners to detect security issues in the target system.

#### 4) Exploitation

When an attacker achieves access due to security weaknesses in a system, then they start to exploit and misuse the resources by collecting sensitive information or disrupting the system availability by demanding money through the use of ransomware malware.

#### 5) Post Exploitation

This phase of social engineering methodology is once the attacker has compromised the system of the victim. At this point, the attacker deals with the collection of crucial relevant information and data. Furthermore, once the attacker knows the security measures of the communication channels, configuration settings, and system networks, the collected data of the target system can be used for continued, future access as per the attacker’s desires. Finally, the attacker cleans the pathways they used and stays invisible by setting up backdoors and rootkits [19].

#### 6) Reporting

Reporting is the final phase, in which the social engineers stop the social engineering cyber-attacks and aggregate the results and documentation.

### C. Life Cycle

#### 1) Investigation

The hackers and cyber criminals first investigate the initial background information like entry points and other weaknesses in the security protocols. In this phase of the SE life cycle, the attacker identifies the victim, gathers background information about the target user, and makes strategies for selecting the attack method.

#### 2) Hook

The attacker attempts to build a relationship of trust with the victim and tries to convince them of what the attacker needs them to believe. The attacker attempts to take control of the interaction as they engage the victim.

#### 3) Play

After building trust with the victim, the attacker exploits the resources available to them and executes the attack on the targeted system to access the information in a timely manner. In this phase, the attacker may disrupt the business or system by siphoning data as well.

#### 4) Exit

The exit is the final stage of the SE life cycle, in which the attacker concludes the interaction without generating distrust. The attacker eliminates all traces of malicious software code and covers their tracks. Errors made by the authentic users who are targeted are much less obvious, making them tougher to recognize than a malware-based intrusion.

### D. Goals

The specific goals of the social engineers are money, ego, revenge, knowledge, and entertainment [20]. They manipulate people into acting differently than they typically do. They want to fool people into providing valuable data and bits of information. Usually, social engineers don’t ever come directly to the victim first. They come to them after gathering information about them or their system, and then they access the target system by fraudulent means. Furthermore, they often establish an immediate connection with the target victim and utilize it as a foundation for building a relationship and an understanding. The attacker uses various approaches for getting relevant information from the victim. Other well-known goals of a social engineer are service disruption, unauthorized access, and financial gain for themselves or another party that hired them [1].

## III. Methodology

Systematic reviews are frequently used in the software engineering domain to summarize the existing literature studies. Garousi *et al.* [22] considered systematic review studies into six types such as; “Systematic Literature Mappings (SLM), Systematic Literature Review (SLR), Grey Literature Mapping (GLM), Grey Literature Review (GLR), Multivocal Literature Mapping (MLM), and Multivocal Literature Review (MLR)”. SLM and SLR based on formal literature and did not include the practitioner’s opinions which have in the grey literature (white papers, website, reports and blogs). Similarly, GLM and GLR based on only grey literature and did not contain the opinion of researchers. However, MLM and MLR contain both formal literature (peer-reviewed journals, conferences and workshops) and grey literature. Detail comparison of the systematic review studies are shown in Table 1 which explicitly illustrate the strength and weaknesses of each systematic review study based on formal and grey literature.

**TABLE 1:** Comparison of Systematic Reviews Based on Formal and Grey Literature

Type of systematic review	Formal literature	Grey literature
SLR	✓	$\times $
SLM	✓	$\times $
GLM	$\times $	✓
GLR	$\times $	✓
MLM	✓	✓
MLR	✓	✓

MLR is more suitable over the other systematic reviews because it is a form of SLR which provides a robust evidence from both researchers (formal published literature; journal, conference and workshop) and practitioners perspective (grey literature; white papers, website, reports and blogs). MLR’s are growing popularity due to bridging a gap between vocal of the industry practitioners and academic researchers. For our current research study, MLR is the most acceptable option because we need information from both formal and grey literature to concisely address our proposed research questions and to identify the current challenges, recommendations, and prospective solutions.

**FIGURE 2.**
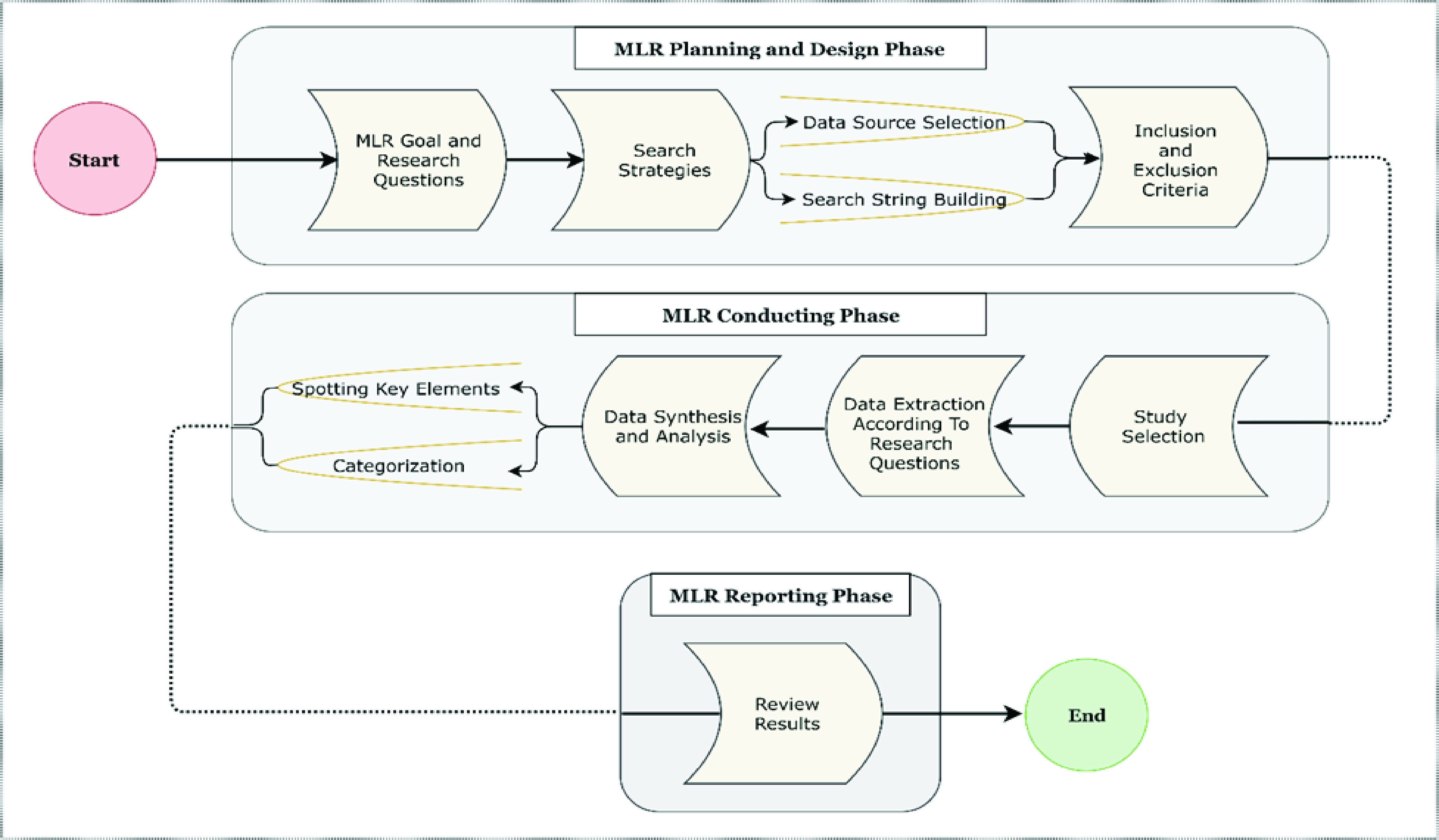
Represents the MLR guidelines from planning, designing, conducting till to the reporting phase.

This research study conducted an MLR based on published guidelines and methods [21]–[24]. MLR is consists of three main phases such as; planning, conducting and reporting as shown in Figure 2.

### A. Research Questions

Our proposed MLR research questions, with brief description, are shown in Table 1.

### B. Data Collection

In this research, we surveyed MRL guidelines and chose those proposed by Garousi *et al.* [22], as shown in Figure 2. An MLR protocol was documented to delineate the complete strategy for the research study. A team of researchers conducted the MLR, and research studies from a time frame from the start of the COVID-19 pandemic until October 2020 were considered. All team members contributed in all phases of the MLR.

### C. Search Strategy

The search string was built by finding keywords and their corresponding alternative words from social engineering studies. Then the designated keywords and their alternative words were chained together with the Boolean operators “AND” and “OR” to express the search string as follows:

“{ *Social engineering OR cyber-threats OR cyber-attack OR online attack OR social attack} AND {Method OR technique OR approach OR platform} AND {COVID-19 OR novel coronavirus OR corona OR coronavirus OR SARS-CoV-2 OR 2019-nCoV} AND {Organization OR sector OR place OR location}”*

The search strategy of MLR necessitates searching both formal and grey literature. In the first stage, the search string was applied to well-known, source-rich digital libraries, such as Scopus and Google Scholar, to find primary studies from the formal literature. In the second stage, the search string was applied to the Google Search engine to find primary studies from the grey literature.

### D. Inclusion and Exclusion Criteria

The following inclusion criteria were used to find related primary studies:

• Studies with a focus on social engineering techniques and methods.
• Studies with a focus on cyber threats/attacks during the COVID-19.
• Studies based on empirical evaluation.

The following exclusion criteria were used to screen out irrelevant primary studies:

• Studies not relevant to the aims of the study.
• Studies written in a language other than English.
• Duplicates and repeated studies.

### E. Quality Assessment Criteria for Formal and Grey Literature

The quality assessment criteria for the formal literature were comprised of six questions, as shown in Table 2. Each question’s score was calculated based on the Kitchenham *et al.* guidelines [24]. The final score was calculated by assigning a 1 for “Yes” and a 0 for “No” for every individual question, with a summation at the end.

**TABLE 2:** Proposed MLR Research Questions With Description

Research Questions	Description
RQ1: Which social engineering techniques are used for the COVID-19 cyber-attacks?	RQ1 explores the techniques that are used during the COVID-19 social engineering–based cyber-attacks, such as phishing, smishing, vishing, pretexting, dumpster diving, extortion, etc.
RQ2: What type of social engineering methods are used for conducting cyber-attacks during the COVID-19 pandemic?	RQ2 attempts to narrow down the types of social engineering methods used for getting access and information, whether social, technical, socio-technical, and physical.
RQ3: Which platform is used as $a$ weapon for social engineering cyber-attacks during the COVID-19 pandemic?	RQ3 is intended to determine the platforms used in COVID-19 cyber-attacks, such as social media, cloud systems, websites, email, etc.
RQ4: What kind of malicious software and attack methods are used for the COVID-19 cyber-attacks?	RQ4 is aimed at identifying the malicious software used, such as ransomware, adware, crimeware, etc., and the attack methods deployed, like impersonation, DoS, DDoS, spoofing, etc.
RQ5: Which sectors and organizations are targeted in COVID-19 cyber-attacks?	RQ5 investigates the targeted organizations and sectors, such as healthcare, hospitals, research, education, banks, etc.
RQ6: What is the economic impact of social engineering cyber-attacks during the COVID-19 pandemic?	RQ6 is the most important questions in the proposed MLR, exploring the financial loss and economic impact of social engineering–based cyber-attacks during the COVID-19 pandemic.

For grey literature, we followed the guidelines for grey literature from Garousi *et al.* [22]. We presented six questions for the grey literature quality assessment criteria, as shown in Table 3. The first tier of grey literature consists of white papers, magazines, government reports, books, and theses. The score for the first tier is equal to 1, a high rank. The second tier of grey literature is comprised of news articles, videos, annual reports, presentations, and websites. The score for the second tier is equal to 0.5, a moderate rank. Finally, the third tier of grey literature contains tweets, blogs, and emails. The score for the third tier is equal to 0, a low rank.

**TABLE 3:** Formal Literature Quality Assessment Criteria

Question No	Criteria	Score
Q1	Are social engineering techniques clearly stated?	Yes=1, No=0
Q2	Are social engineering types clearly stated?	Yes=1, No=0
Q3	Are the social engineering cyber-attack platforms during the COVID-19 specified?	Yes=1, No=0
Q4	Are the social engineering cyber-attack types during the COVID-19 mentioned?	Yes=1, No=0
Q5	Are the target organizations and sectors for social engineering cyber-attacks during the COVID-19 cited?	Yes=1, No=0
Q6	Are the economic impacts of social engineering cyber-attacks during the COVID-19 mentioned?	Yes=1, No=0

### F. Study Selection

**FIGURE 3.**
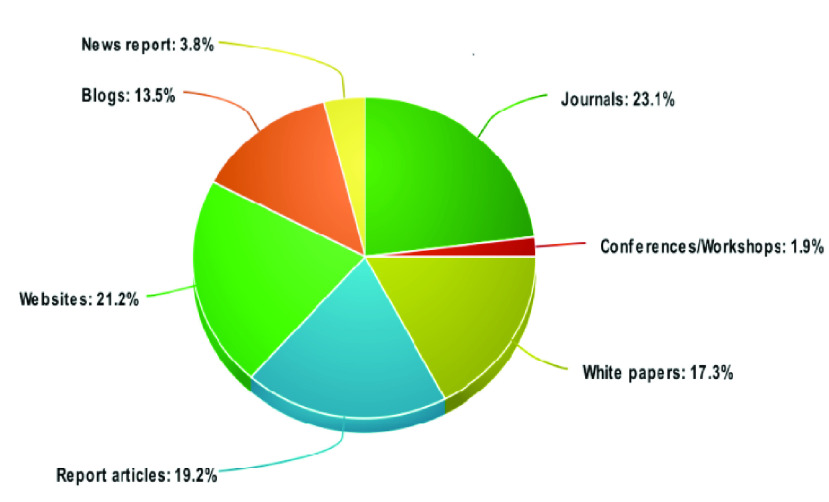
Selected article distributions over various sources from both formal and grey literature.

**FIGURE 4.**
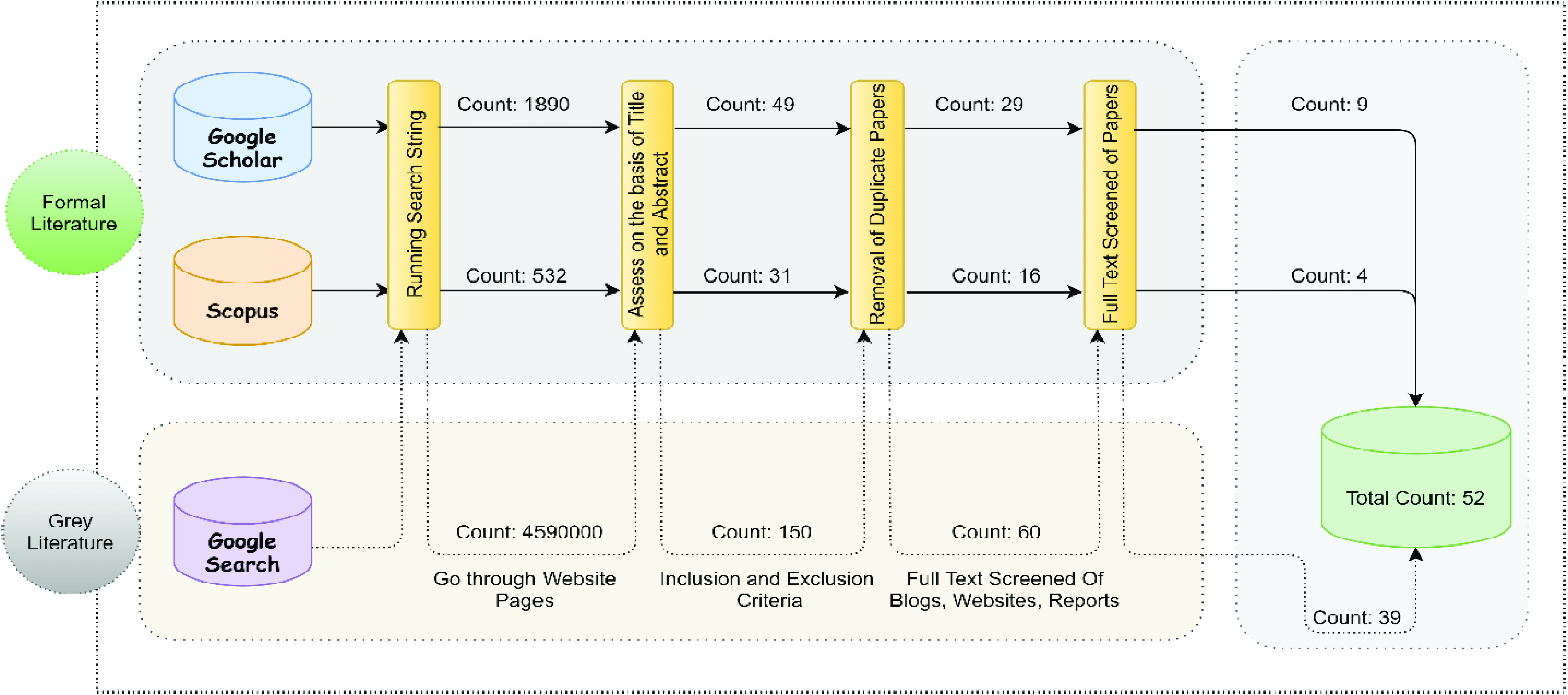
MLR study selection process from google scholar, scopus and google search engine by applying inclusion/ exclusion and quality assessment criteria.

The study selection was comprised of both formal and grey literature from Scopus, Google Scholar, and Google Search engine. Figure 3 shows the distribution of formal and grey literature studies from various sources. The complete MLR study selection procedure is shown in Figure 4.

#### 1) Formal Literature Selection

In the initial phase, we identified 532 results from Scopus and 1,890 from Google Scholar that were relevant to our proposed research topic. By analyzing the titles, abstracts, and keywords of the papers according to our inclusion and exclusion criteria and removing duplicate papers, the number of papers were reduced to 16 for Scopus and 29 for Google Scholar. By studying the full text of those papers, we finally selected a total of 13 papers from both Scopus and Google Scholar.

#### 2) Grey Literature Selection

We used the Google Search engine to locate grey literature. In the initial search, we found 4,590,000 results, as listed on the top results page. By limiting the search to only the first 15 pages [25], due to the collection of more information from Google Search engine. We applied the inclusion and exclusion criteria to the titles and keywords, the number of sources was reduced to 60. Further screening the full texts for relevancy to our topic, we finally selected 39 articles in the form of websites, blogs, reports, news reports, and white papers.

### G. Data Extraction

To answer our research questions, we identified and extracted the relevant information and data by employing the predefined data extraction procedure of MLR guidelines. The collected data are stored in Microsoft Excel spreadsheets for evaluation by including the title, author name, SE technique, SE types, SE methods, SE platform used, type of malicious software used, targeted organizations and sectors, and the year of published articles. Appendix A and Appendix B shown the collected data, including their quality assessment scores for each research questions, along with study title, author name, and year.

### H. Data Synthesis and Analysis

In the data synthesis phase of the study, the primary studies were carefully evaluated in order to describe the final results. The information and data were collected in the extraction phase, and they were further analyzed to address our research questions and help us draw the conclusions of the proposed study.

## IV. Results

### A. Social Engineering Techniques Used During the COVID-19 Pandemic (RQ1)

Numerous social engineering techniques were used by scammers, hackers, and cybercriminals for cyber-attacks with an objective to exploit the victim’s systems.

According to our research regarding social engineering techniques, phishing is the most common techniques used by the threat actors at 35%. Email platforms were used as a weapon for leading phishing attacks by using various misleading email links and fake news. Spam is the second highest used social engineering technique, at 16%. Scams were the third most common technique at 14%. For example; scams include contents like; loan emails, COVID-19 tests news, bogus insurance invoices, employment news etc. Moreover, the attackers also used smishing and vishing techniques during the COVID-19 pandemic by sending text messages to and calling mobile numbers, WhatsApp users, and other social media accounts to trick victims, and both techniques combined account for nearly 22% of the overall weightage. Finally, the other techniques such as; spear-phishing, extortion, cyberbullying, cyber-stalking, pre-texting, and fear-attacks were executed much less frequently.

### B. Type of Social Engineering Methods Used for Conducting Cyber-Attacks During the COVID-19 Pandemic (RQ2)

There are four types of methods used by threat actors for conducting cyber-attacks. In the COVID-19 pandemic cyber-attack scenarios, they used the socio-technical method 44% of the time. The hackers also used the technical method to forcibly attack the victims’ systems to get the desired information in 29% of cases. The social method, such as texting or calling the victims and using fake identities to get relevant information about the victims, was also used a total of 23% of the time during the COVID-19 pandemic. Finally, the physical method was also used in a very small amount of cases, only 4%. The overall percentages for the four methods are shown in Figure 6.

**FIGURE 5.**
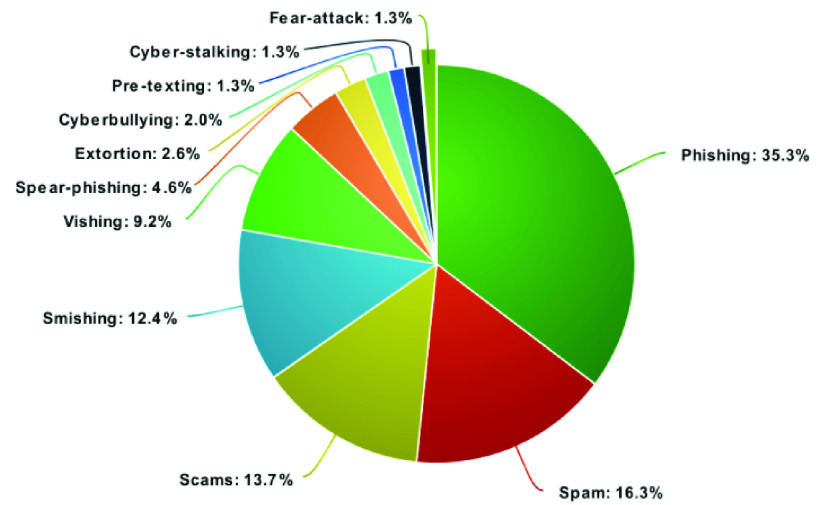
Different social engineering techniques used for cyber-attacks/threats during the COVID-19 pandemic shown in percentages of the attacks/threats.

**FIGURE 6.**
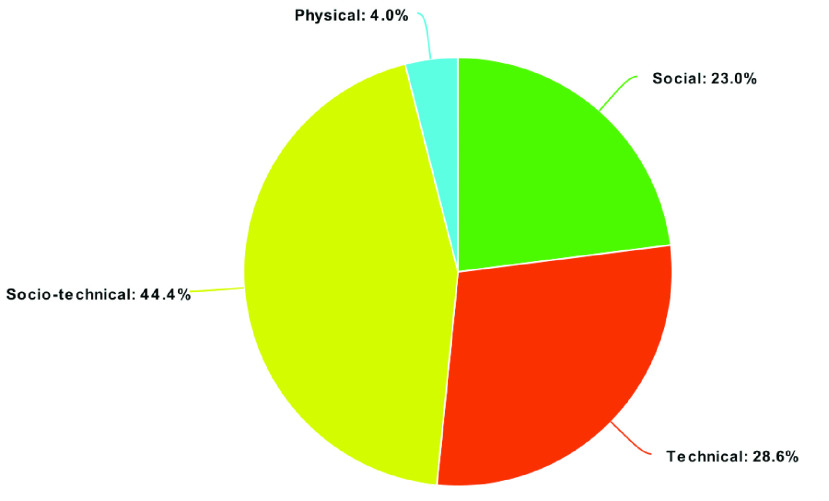
Social engineering types used for cyber-attacks/threats during the COVID-19 pandemic by percentage of attacks/threats.

### C. The Platforms Used as Weapons for Social Engineering–Based Cyber-Attacks During the COVID-19 Pandemic (RQ3)

**FIGURE 7.**
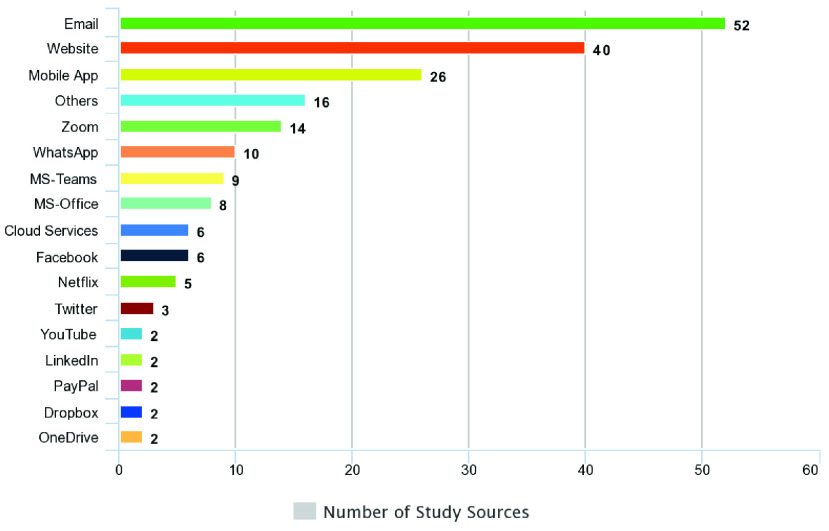
Mapping of study sources by the platform used as a weapon for social engineering based cyber-attacks/threats.

Figure 7 shows the platforms used by the attackers for performing social engineering cyber-attacks/threats. Email is the most used platform by a wide margin and is discussed by 52 studies. This correlates exactly with RQ1, in which the top used technique was phishing done mainly via emails. The attackers and cybercriminals also developed fake websites related to the coronavirus with news and data intended to trick users, which is the second most used platform. Similarly, the attackers also developed various mobile applications for coronavirus updates to target the user for accessing and getting information.

The majority of mobile devices that were hacked during the COVID-19 pandemic were targeted with the use of fake applications from the threat actors. Due to the coronavirus, many organizations moved their activities online, and they mostly used platforms like Zoom and Microsoft Teams for online meetings and video conferencing, which have also been hacked many times during the COVID-19 pandemic. Furthermore, WhatsApp as a primary source of communications, was hacked several time times. Other well-known social media platforms were also hacked and used as weapons for conducting social engineering cyber-attacks, as shown in Figure 7.

### D. Kinds of Malicious Software and Attack Methods Used for Social Engineering Cyber-Attacks/Threats (RQ4)

**FIGURE 8.**
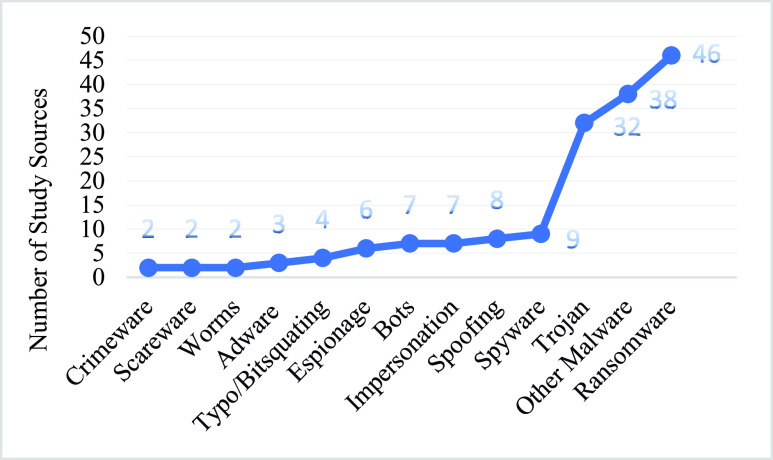
Growth trends in malicious software used in social engineering based cyber-attacks/threats by number of study sources citing them.

Figure 8 shows the growth trends of social engineering cyber-attacks/threats using different malicious software. Ransomware is the most cited malicious software used for cyber-attacks on various public and private sector organizations during the COVID-19 pandemic. The generic “Other Malware” category is the second most cited, as shown in Figure 8, consisting of various malicious software and cyber-attack methods, such as e-skimming, cryptominer software, BEC, DoS, brute-force attempt, DDoS, cyber-sabotage, and malicious URL attacks have also been conducted during the COVID-19 pandemic. Trojan malware was also used in significant amounts. Spyware, spoofing, impersonation, and bots were used at a moderate level, compared to the other top-cited categories.

**FIGURE 9.**
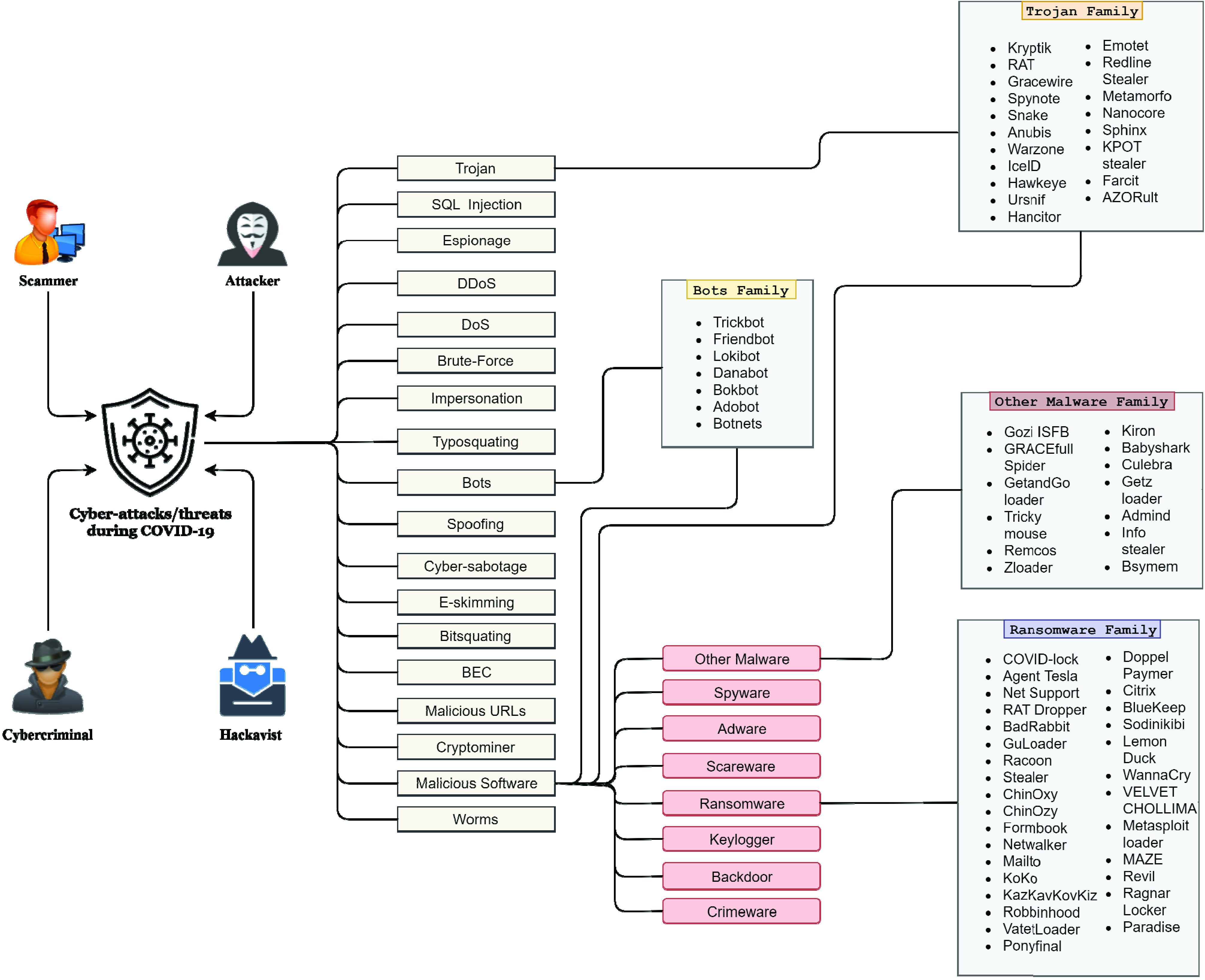
Detailed overview of the malicious software and their sub-families used in social engineering based cyber-attacks/threats during the COVID-19 pandemic.

Three types of malicious software were the most commonly used, ransomware, trojans, and bots, as shown in Figure 9 with their specific deployments and families. By count of these unique family, ransomware was used the most with 30 families, trojans second with 19 families, and finally bots third with 7 families. The generic “Other Malware Family” includes 13 families, as presented in Figure 9.

The trojan families that were used the most during the COVID-19 Pandemic are; RAT, AZORult, Emotet, KPOT, Nanocore, and Sphinx.

Emotet was used mostly for banking and financial cyber-attacks. Similarly, Netwalker, MAZE, Stealer, Maillot, Covid-lock, Dopper-paymer, and Agent Tesla are ransomware family that was widely used as a threat for demanding money and financial benefits. Furthermore, from the bot family type, Loki-Bots was highly used, and Spider, Remcos, and Info-Stealer from the other malware family were regularly used during the COVID-19 pandemic for cyber-attacks /threats.

### E. Mostly Targeted Organizations and Sectors During COVID-19 Cyber-Attacks (RQ5)

Cybercriminals exploit various organizations and industries during the COVID-19 pandemic, such as healthcare, hospitals, private and public sectors, government institutions, banking, and finance. The top targeted organizations are healthcare companies and hospitals due to their weak security setups. The targeting of healthcare organizations carried out by the advanced cyber hackers and attackers.

### F. The Economic Impact of Social Engineering Cyber-Attacks During the COVID-19 Pandemic (RQ6)

The economic impact of social engineering cyber-attacks is rising exponentially with the advancement and general use of new technologies. According to Accenture’s annual security report, security breaches increased 67% in the past five years, and in the last year, companies spent $110 billion worldwide for protection against cyber-attacks [26].

During the COVID-19 pandemic, the University of California San Francisco School of Medicine was targeted by hackers with ransomware, and they paid $1.14 million to remove the ransomware [27]. Infosecurity Magazine mentioned that the UK’s National Fraud and Cybercrime Reporting Center claimed that online scams had captured 16,352 victims through auction schemes and online shopping during the COVID-19 pandemic and lost approximately £17 million [28]. During the COVID-19 pandemic, two reports from Australia and the US stated that the Australian Competition and Consumer Commission’s Scam Watch reports over 2,700 scams causing losses of $16,390,650 AUD and that the Federal Trade Commission of the US estimated that $12 million USD were lost in fraudulent activities [40]. During the COVID-19 pandemic, Wiggen [30] reported that Russian malware targeted Ukraine, encrypting crucial data from computer systems and making it useless; the cost of the damage was estimated more than $10 billion.

According to at least one prediction [31], the Global Cybersecurity Market will total $152 billion USD by 2025 because of the growing concern over cyber-attacks/threats and data breaches that are confronting organizations.

## V. Discussion

This review has described social engineering cyber-attacks/threats on organizations and critical infrastructure during the COVID-19 pandemic. Throughout this review, we identified social engineering techniques; applied methods; platforms, malicious software, and attack methods used; and finally, the organizations targeted. Information on our proposed research questions is available more in grey literature sources than formal literature, demonstrating that practitioners are more active in providing social engineering–based cyber solutions and solving security issues as shown in Figure 11. The types of malicious software that have been used was the research question most cited and mentioned because it involves a wide variety of malicious software, relevant software families, and attack methods. Therefore, more research and cyber solutions are needed to address cyber-attacks and threats that come in the form of malicious software. We must secure social media and other communications platforms as well, due to their usage as a weapon for different cyber-attacks. Platforms of cyber-attacks/threats are the second most cited research question in both formal and grey literature, as shown in Figure 11.

**FIGURE 10.**
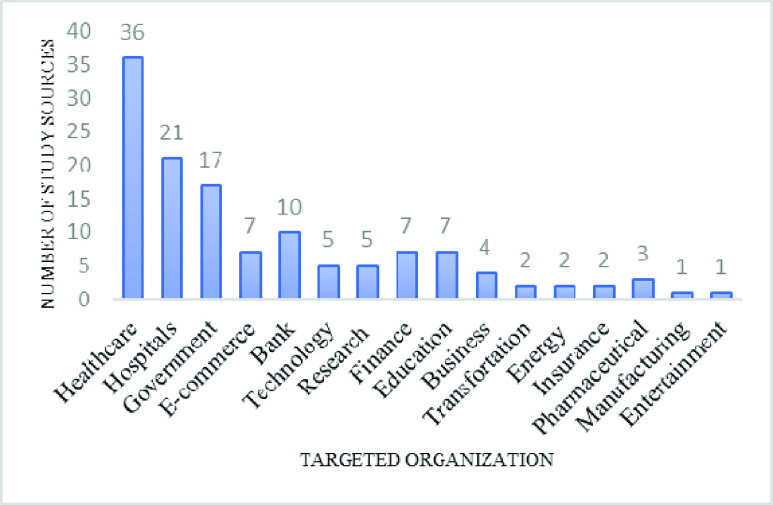
Breakdown of the study sources by type of organization targeted with social engineering based cyber-attacks/threats.

**FIGURE 11.**
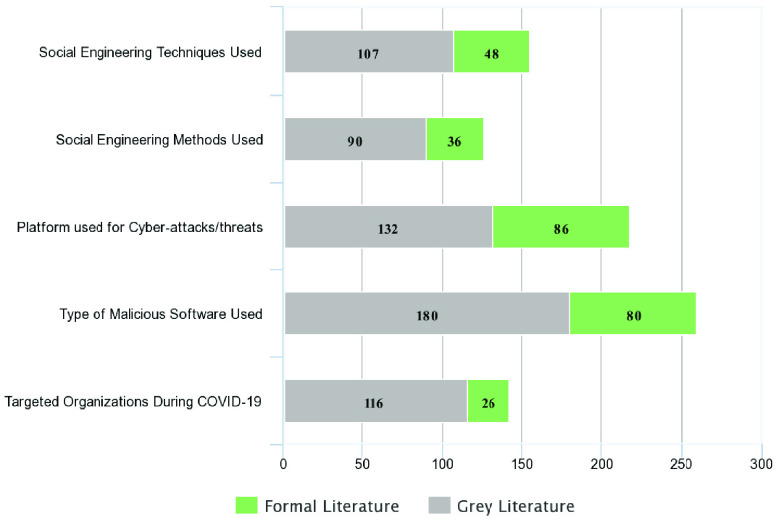
Prevalence of research questions among formal and grey literature sources.

Similarly, we also explored the economic impacts of social engineering cyber-attacks with recent estimates and a future projection through 2025. Cyber solutions need to be robust and consistent because of the increasing numbers of cybercriminals, hacktivists, scammers, and extortion groups using different social engineering techniques to exploit critical assets and systems. Phishing attacks were used in different forms, such as spear-phishing, smishing, and vishing, via emails, calls, and text messages. These cyber-attacks can be reduced with awareness campaigns and by applying security email spam filters.

Consequently, solutions to social engineering–based cyber-attacks/threats require a high level of innovation, teamwork, collaboration, and performance. Cyber solutions need to be adaptive and generalizable to various organizations, especially for the healthcare industry and hospitals that are ripe targets for threat actors these days. Social engineering–based cyber solutions require significant research and development to produce outcomes capable of instant incidence response in the case of unexpected and surprising cyber events. Our review is based on the perspectives of both researchers and practitioners and will benefit both academia and industries in carrying out initial assessments for their own research and development.

## VI. The Motivation Behind Social Engineering Cyber-Attacks/Threats

### A. Challenges

**FIGURE 12.**
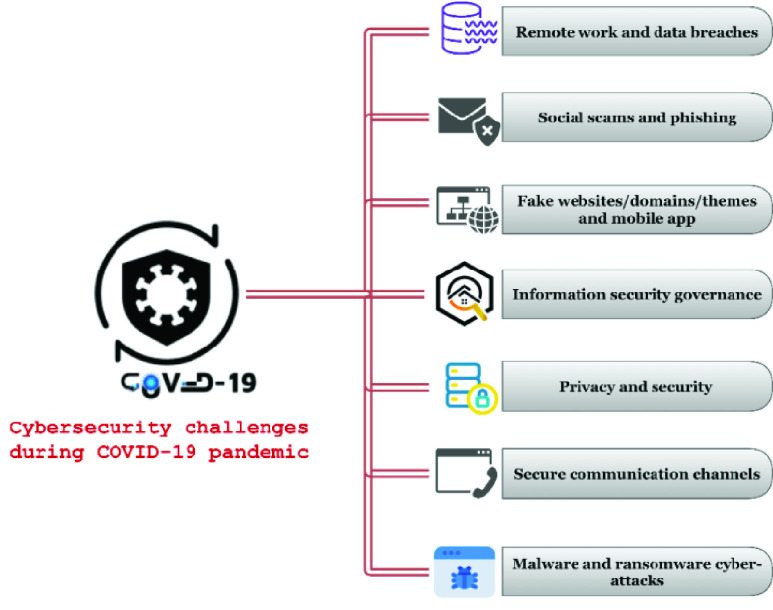
Cybersecurity challenges during the COVID-19 pandemic.

The swift circulation of COVID-19 created potential cybersecurity challenges that need to be addressed to protect victims and critical infrastructure. Our MLR explored several cybersecurity challenges during the COVID-19 pandemic, and after the authors’ careful observations and research, we divided these challenges into seven main categories, as shown in Figure 12.

#### 1) Remote Work and Data Breaches

Remote working allows geographically spread out employees to work from various locations to fulfill their assigned tasks. The nature of office work has largely been transferred to remote working spaces due to the COVID-19 pandemic, and the majority of large organizations proceed with their work remotely from home via online platforms. However, the remote working present challenges and provides disclosures for a broad spectrum of social engineering cyber-attacks and cybersecurity issues through emails, file sharing, and access to networks via user devices [32]. In more than 12 countries, 3,000 employees were surveyed; 94% of them suffered from data breaches via cyber-attacks, with an average number of 2.17 breaches each [33]. Home networks remain less secure compared to organizational internal networks, possibly posing greater dangers for employees already at a larger risk of cyber-attacks. Also, a large number of people are not trained to work remotely in a secure way. A report from the International Association of IT Asset Managers (IATAM) is cautioning that working from home during the COVID-19 pandemic is allowing for plentiful data breaches [34], [35].

#### 2) Social Scams and Phishing

Phishing attacks and scams during the COVID-19 pandemic started in January 2020 and disseminated very quickly, even producing thousands of fake sites and scams every day. UK regulatory authorities noticed a surge in the registration of new webpages related to the COVID-19 pandemic which seems suspicious as threat vectors for exploitation and cyber-attacks [36].

Scams are more prevalent and costly due to the financial situation of most people during the COVID-19 pandemic, as those suffering from income loss and joblessness come under threat from scams. Similarly, scammers further target vulnerable people by posting fake advertisements and news regarding treatment of the coronavirus and vaccines [37]. In these efforts, fraudsters use software tools for scamming and phishing and use subcategories of these techniques, such as spear-phishing, smishing, and vishing. They use different platforms like emails, texts, social media posts, and robocalls, for impersonation schemes [38].

#### 3) Fake Websites, Domains, Themes, and Mobile Apps

Attackers and cybercriminals continue to build fake websites and mobile apps to steal credentials relevant to financial assistance and personal identification. The threat actors develop themes and website templates that mimic the government and trusted non-governmental organizations, such as the World Health Organization, Internal Revenue Service, and Centers for Disease Control [39].

A statistical report from Palo Alto researchers [40] through the end of March 2020 showed that a total of 116,357 new domain titles and registrations related to COVID-19 were made during that time. They elaborated, “Out of these, 2,022 are malicious and 40,261 are with high-risk.”

#### 4) Privacy and Security

Numerous organizations and governments have worked on efforts to develop track-and-trace mobile and web applications to empower society to get back to normal and avoid the spread of the COVID-19. Similarly, the rise of digital world services originates at the cost of privacy. However, there is a need for the right balance between institutional response, user access, and information privacy. The use of drones during the COVID-19 pandemic may also violate privacy if the data is stored or transmitted in the form of images and videos. Similarly, cybersecurity-related issues, such as brute force attacks, injections, eavesdropping, replay attacks on the communication channels, and storage drain attacks, need to be addressed to protect end users from various cyber-attacks. These privacy and security challenges are causing researchers and practitioners to re-think the application of digital transformation initiatives [62].

#### 5) Information Security Governance

Organizations need to understand where their approaches to information security are truly symbolic. It is essential for organizations to adopt the “Digital Security Governance” for their existing security approaches. Digital Security Governance is the “practitioners and decision makers by providing a deeper understanding of how organizations and their security approaches are actually affected by digitalization” [42]. The sharing of information by organizations needs to be in accordance with the legal and regulatory authorities as well as digital laws because data can be critical when it is related to business, industry, and personal lives. Software tools should be developed for information mapping according to standard policy and supporting security measures. Research should be performed on where the information of an organization is accessed and by whom and what the existing platforms that generate, process and store information are. Is it in accordance with reasonable security standards or not?

#### 6) Secure Communication Channels

Effective and secure digital communication channels are needed, and they are even more critical during pandemic crisis management and onwards. The disseminated workforce needs secure communication channels to carry out their tasks in a consistent, accurate, and safe manner. Security standards are necessary for organizations to effectively communicate with their employees and to monitor these means of communication for potential security vulnerabilities. Cybersecurity efforts are essential for improving and securing digital devices and networks for promoting business continuity. It is crucial to establish device security because most wearable devices and the “internet of things” (IoT) are also vulnerable to cyber-attacks [43].

#### 7) Malware and Ransomware Cyber-Attacks

Attackers use different malware and ransomware for resource and system exploitation, as shown in Figure 10, and target critical infrastructures, such as healthcare organizations, hospitals, and banks, for financial gain. Threat actors generally use phishing techniques for a ransomware attack to inject malware code into the victim’s computer and network system in order to encrypt it and make the data inaccessible to the victim. The threat actor then tries to extort a monetary payment from the victim in exchange for the key required to decrypt the compromised information files and data. For example, a British research company that was preparing the COVID-19 vaccines to conduct trials was attacked with MAZE ransomware [65]. Cybersecurity experts and researchers need to develop robust software tools for penetration testing, guidelines, and security standards to detect and comprehend the threat landscape and potential cybersecurity vulnerabilities.

### B. Recommendations

Social engineering–based cyber-attacks targeted a diversity of victims from secure and intricate organizations to single individuals. The main objective of our proposed recommendations is to protect victims from different kinds of cyber-attacks at the initial level and how to mitigate them. These recommendations can be measured as the most minute level of defense for organizations and for end users as well. The following are the proposed recommendations:

• Individuals must use strong password practices and apply multi-factor password authentication for accessing their own social media accounts as well as remote devices to limit cyber-attacks from exploiting data breaches and stealing information.
• Organizations must implement user access restrictions and control mechanisms for remote workers to protect them from accessing sensitive data and information and to provide them access only based on their job responsibilities. This will significantly reduce the influence of social engineering cyber-attacks.
• Back up all critical information and data in a consistent manner and keep it safe in an external system, external hard drive, or in a secure cloud storage providers.
• Be conscious of suspicious messages with spelling errors, suspect emails, pop-up advertisements with fake offers, news regarding corona vaccines and treatment, and private and public financial offers. The official authorities never use personal email addresses for sending such information. Always trust and rely on well-known governmental organizations and NGOs for information updates during a pandemic, such as the World Health Organization, the Centers for Disease Control, and the National Institutes of Health.
• Regarding fake websites, themes, domains, and mobile apps, double-check lookalike domains, spelling errors in website headings, and top content information. Authenticate the company’s legal website before entering login credentials and other sensitive information.
• Be aware of the common social engineering cyber-attacks and threats such as robocalls, phishing, smishing, and vishing and how hackers and cybercriminals target victims by triggering fears of losing access to private data and money.
• Always review the policies on privacy and security of different software’s when using it in a remote work environment for conference meetings and telehealth.
• Avoid clicking on suspicious links received from unknown sources that may redirect you to the malicious software and suspicious files to download it on your device and computer systems in the form of coronavirus app, antivirus, etc. Keep your computer and devices security, firewall, and software’s up to date.

### C. Prospective Solutions

#### 1) Training and Awareness

Organizations’ cybersecurity teams, whether their own or third-party hires, must stay focused on detection technologies for the stream of traffic initiating from remote employees. Similarly, the cybersecurity teams must provide an initial level of security awareness for all employees, such as the use of a strong password, secure sharing of data and information, software updates, cookies and session hijacking, detection of malicious URLs, home-based network and router security, protection security for the IoT and wearable devices, and other relevant educational awareness training. More specifically, to educate the remote employees on incident awareness and management to support their cybersecurity teams and improve response times during cyber-attacks/threats. This can be done via simulations of social engineering–based cyber-attacks with remote employees to teach them how to detect, respond, and recover in time.

#### 2) Artificial Intelligence

Artificial intelligence uses machine-learning algorithms on various datasets to perform statistical analysis, allowing for the making of assumptions about behavioral patterns. Algorithms adjust and perform functions according to their programmed purpose and learn from the applied data. According to future predictions, up to 70% of organizations will be adopting artificial intelligence in the domain of cybersecurity [67]. Artificial intelligence–based tools play a significant role in understanding and predicting cyber-attacks/threats. A recent survey report from Webroot [44] included 800 respondents among information technology professionals with cybersecurity decision-making powers from Australia, New Zealand, Japan, the US, and the UK. They revealed that 96% of the survey respondents are using artificial intelligence and machine learning tools for cybersecurity. Artificial intelligence systems are currently used for traffic pattern and behavioral detection of zero-day cyber-attacks and continue to progress through self-learning, and generating results more quickly and more precise than analysts [45], [46]. Artificial intelligence can improve security performance and predictions of cyber-attacks/threats, malware, trojans, and botnets [47].

#### 3) Big Data Analytics and Cyber Resilience

Cybersecurity attacks are increasing with the emergence of technology, and cybercriminals are using various social engineering and other sophisticated techniques to exploit victims. Various organizations and individuals are suffering cybersecurity attacks and security breaches on a massive scale, especially during the COVID-19 pandemic. Data analytics play a prominent role in leveraging cyber resilience and assist in mitigating and reducing cyber threats and crimes [48]. Big data analytics reviews an enormous amount of data from different historical cyberattacks and can help analysts assess and detect anomalies within computer systems and networks to protect the system from possible future cyber-attacks/threats [49], [50]. Using big data analytics with different correlation algorithms for anomaly detection in combination with strong cybersecurity principles can assist organizations in enhancing their cyber resilience [51]. Big data analytics can be significant for accumulating all historical data from cyberattacks and threats related to the COVID-19 pandemic in order to forecast future cyber-threats.

#### 4) Blockchain and the Internet of Things

Wearable and IoT devices are growing very quickly due to current advancements in technology. However, they are vulnerable to cyber-attacks as well, and these devices need to be secured by protecting sensitive information and user’s personal data. One way of protecting these devices is to implement the concept of blockchain technology. Blockchain is a distributed network used by millions around the globe. In the blockchain, the data of these devices can be added but not copied or changed, and only managed through the use of a computers cluster which is not owned by any single person [52]. By applying the blockchain technology to healthcare IoT and other critical infrastructure, the recent growth in cyber-attacks/threats and theft of information in the age of the COVID-19 pandemic can be reduced. Blockchain technology is stepping up to overcome security apprehensions in the appearance of current cybersecurity breaches [53].

## VII. Limitations of the Study

One of the limitations of the MLR might be the subjective decisions and search terms used for data extraction process from the grey and formal literature, particularly the use of the three core search engines of Google Search engine, Scopus, and Google Scholar, which may lead to missing some studies in the results. This effect was reduced by imposing the search term limitations and with the use of alternative keywords and repetitions of the search terms. Similarly, the subjectivity of our decisions was further decreased by the authors’ detailed, repeated reviews. Grey literature sources signify the voice of practitioners in the real industrial environment. Another possible limitation specifically for the grey literature, however, is that few of the practitioners’ recurring opinions overlap with those of other practitioners. To mitigate this effect, we drew our data from reputable reports, blogs, website, whitepapers, and magazines based on our defined quality assessment criteria.

## VIII. Conclusion and Future Work

To the best of our knowledge, no systematic MLR has focused on the emerging social engineering–based cyber-attacks /threats from both the researchers’ and the practitioners’ perspectives. The COVID-19 pandemic has caused a considerable and long-lasting social and economic impact on the world, and social engineering–based cyber-attacks/threats are one of the primary motives for the present insecurities. Social engineering–based cyber-attacks are based on psychological and systematic techniques to manipulate users that cannot be controlled solely through the use of technology.

The objective of this research study was to detect the state-of-the-art and state-of-the-practice social engineering–based techniques, attack methods, and platforms used for conducting successful cyber-attacks/threats with economic and social impacts on various organizations. This review highlighted the most targeted organizations and critical infrastructure that are exploited by cybercriminals during the COVID-19 pandemic. This research work provided an MLR related to the rise of social engineering–based cyber-attacks/threats since the emergence of the COVID-19 pandemic. In total, 52 primary studies were selected from both formal and grey literature based on published guidelines for conducting an MLR. The review revealed that some of the major social engineering–based techniques used during the COVID-19 pandemic are phishing, scamming, spamming, smishing, and vishing in combination with the mostly used socio-technical methods of fake emails, websites, and mobile apps used as weaponized platforms for conducting cyber-attacks. Finally, the potential economic impacts of successfully conducted cyber-attacks on various organizations and critical infrastructure were also discussed. Most significantly, we explored open challenges, general recommendations, and prospective solutions by using the latest technology.

From the conducted MLR, several future works were identified that will support security practitioners and researchers in addressing the proposed challenges relevant to cybersecurity by applying their research and development skills to propose new tools, security standards, policies, and frameworks in combination with the use of emerging technologies, such as artificial intelligence, blockchain, and big data analytics. In the future, we intended to propose a framework for training and awareness aimed at the initial level of cybersecurity awareness for organizations and end-users.

## Appendix A

See Table 5.

**TABLE 4:** Grey Literature Quality Assessment Criteria

Question No	Criteria	Score
Q1	Is the organization reputable regarding social engineering cyber-threats/attacks?	1^st^ tier = High = 1, 2^nd^tier = Moderate =0.5, 3^rd^ tier = Low = 0
Q2	Does the author have expertise in social engineering?	1^st^ tier = High = 1, 2^nd^tier = Moderate =0.5, 3^rd^ tier = Low = 0
Q3	Does the source clearly mention the social engineering techniques and types used for cyber-attacks?	1^st^ tier = High = 1, 2^nd^tier = Moderate =0.5, 3^rd^ tier = Low = 0
Q4	Are the social engineering platforms used and organizations targeted for cyber-attacks clearly stated with a date?	1^st^ tier = High = 1, 2^nd^tier = Moderate =0.5, 3^rd^ tier = Low = 0
Q5	Does the work cover one or more research questions?	1^st^ tier = High = 1, 2^nd^tier = Moderate =0.5, 3^rd^ tier = Low = 0
Q6	Are the economic impacts of social engineering cyber-attacks during the COVID-19 stated?	1^st^ tier = High = 1, 2^nd^tier = Moderate =0.5, 3^rd^ tier = Low = 0

**TABLE 5:** List of Formal Literature After Quality Assessment Criteria

S.No	Paper Title	Journal/Conference	Year	Author	RQ1	RQ2	RQ3	RQ4	RQ5	RQ6
1	“A multi-level influence model of COVID-19 themed cybercrime”	Journal	2020	Naidoo [54]	1	1	1	1	1	1
2	“Coronavirus Social Engineering Attacks: Issues and Recommendations”	Journal	2020	Alzahrani [55]	1	0	1	1	1	0
3	“Are your IT staff ready for the pandemic-driven insider threat?”	Journal	2020	Chapman [56]	1	1	1	0	0	1
4	“Have You Been a Victim of COVID-19-Related Cyber Incidents? Survey, Taxonomy, and Mitigation Strategies”	Journal	2020	Imran et al. [57]	1	1	1	1	1	1
5	“Beyond the Virus: A First Look at Coronavirus-themed Mobile Malware”	Journal	2020	He et al. [58]	1	1	1	1	0	0
6	“Cyber Security in the Age of COVID-19: A Timeline and Analysis of Cyber-Crime and Cyber-Attacks during the Pandemic”	Journal	2020	Lallie et al. [59]	1	1	1	1	1	1
7	“Don’t Fish in Troubled Waters! Characterizing Coronavirus-themed Cryptocurrency Scams”	Journal	2020	Xia et al. [60]	1	1	1	1	0	1
8	“IT Risk and Resilience—Cybersecurity Response to COVID-19”	Journal	2020	Weil and Murugesan [62]	1	1	1	1	1	0
9	“Randomized Cyber Attack Simulation Model: A Cybersecurity Mitigation Proposal for Post COVID-19 Digital Era”	Journal	2020	Okereafor and Adelaiye [62]	1	1	1	1	0	1
10	“Recommendations for Ordinary Users from Mitigating Phishing and Cybercrime Risks During COVID-19 Pandemic”	Conference	2020	Chi Tran [63]	1	1	0	1	0	0
11	“Tackling the Cybersecurity Impacts of The Coronavirus Outbreak as a Challenge to Internet Safety”	Journal	2020	Okereafor and Adebola [64]	1	1	1	1	1	0
12	“The impact of COVID-19 on cyber-crime and state-sponsored cyber activities”	Journal	2020	Johannes Wiggen [30]	1	1	1	1	1	1
13	“Ten Deadly Cyber Security Threats Amid COVID-19 Pandemic”	Journal	2020	Khan et al. [65]	1	1	1	1	1	0

## Appendix B

See Table 6.

**TABLE 6:** List of Grey Literature After Quality Assessment Criteria

S.No	Topic Title	Year	Organization/Author/Report, Blog, Whitepaper, News Report, Magazines	RQ1	RQ2	RQ3	RQ4	RQ5	RQ6
14	“Situational Awareness: Cyber Threats Heightened by COVID-19 and How to Protect Against Them”	2020	Adan Meyers/Blog [66]	1	1	1	1	1	0
15	“Developing Story: COVID-19 Used in Malicious Campaigns”	2020	TrendMicro/Blog [67]	0.5	0	1	1	1	0
16	“Flattening the Scam Curve: Be Prepared for Uptick in COVID-19 Social Engineering Cyber Attacks”	2020	Daniels and Oberly/white paper [68]	1	1	1	1	1	0
17	“Criminals exploit COVID-19 fears to launch ‘unprecedented wave’ of global cyberattacks”	2020	Arabnews/website [69]	0.5	0.5	0.5	0.5	0.5	
18	“COVID-19 Exploited by Malicious Cyber Actors”	2020	Department of homeland security and Cybersecurity and Infrastructure Security Agency/website (report) [70]	1	1	1	1	1	0
19	“How to protect your companies from rising cyber-attacks and fraud amid the COVID-19 outbreak”	2020	PWC (US)/Website [71]	0.5	0	0	1	0.5	0
20	“43 COVID-19 Cybersecurity Statistics”	2020	Pandasecurity.com/mediacenter/Article Report [73]	0.5	0	0.5	1	1	1
21	“Exploiting a crisis: How cybercriminals behaved during the outbreak”	2020	Microsoft/Report [73]	1	1	1	0.5	0.5	0
22	“Cyber Criminals are Leveraging Coronavirus to Boost Profit”	2020	LIFARS/website [74]	0.5	0	0.5	0.5	0	0
23	“Cyber Security in Uncertain Times”	2020	Veridk/Security expert views/website [75]	0.5	1	0.5	0.5	0	0
24	“Ransomware groups continue to target healthcare, critical services; here’s how to reduce risk”	2020	Microsoft/Blog [76]	1	1	0.5	1	1	0
25	“Threat Actors Exploit Covid-19 in Cyber Crime Campaigns”	2020	LookingGlass/Blog [77]	0.5	0	0.5	0.5	1	0
26	“Social Engineering Attacks And COVID-19”	2020	AON/Website [79]	0.5	0.5	0.5	0.5	0.5	0
27	“Why the coronavirus pandemic presents a golden opportunity for hackers”	2020	Security Info Watch.com/Lucas Ropek/Website (report) [79]	0.5	0.5	0	0.5	0.5	0
28	“Bad Actors Have Adapted Well to the Pandemic Crisis”	2020	Government Technology/Lucas Ropek/Report [80]	1	1	0.5	0.5	1	0
29	“Latest Covid-19-related cyber security news: Hospitals under attack”	2020	F-Secure/Jason Satler/Blog [81]	1	1	0.5	0.5	1	1
30	“COVID-19 Social Engineering Attacks”	2020	CSO/US/Report article [82]	1	1	1	0.5	1	0
31	“Types of Social Engineering Attacks: Detecting the Latest Scams”	2020	BioCatch/Blog [83]	0.5	0	0.5	0.5	1	1
32	“Coronavirus: Its Four Most Prevalent Cyber Threats”	2020	Security Boulevard/Pascal Geenens/Report article [84]	1	1	0.5	0.5	1	1
33	“Security Experts Battle Hackers, COVID-19 Cyberattacks”	2020	SDX Central/Jessica Lyons Hardcastle/Report article [85]	1	1	0.5	0.5	1	0
34	“Coronavirus: Cyber-attacks on banks seen spiking, says Carbon Black”	2020	ComputerWeekly.com/Alex/Website [86]	0.5	1	0.5	1	0.5	0
35	“Trend Micro: COVID-19 related malware and spam on the rise”	2020	Security Brief/Shannon Williams/Website [87]	1	1	0.5	0	0.5	0
36	“Ransomware Gangs and COVID-19 Cyberattacks Dominate the Threat Landscape”	2020	INSIGHTS/Kevin Deffily/Blog [88]	0.5	1	0.5	1	0.5	0
37	“Cyber attacks in the pandemic era: More of the same?”	2020	Techerati/Lavi Lazarovitz/Website [89]	0.5	0.5	0.5	1	1	0
38	“Live Updates: COVID-19 Cybersecurity Alerts”	2020	CYWARE/Website [90]	0.5	0	1	1	1	1
39	“Cybersecurity’s dual mission during the coronavirus crisis”	2020	McKinsey & Company/Boehm et al./Report article [91]	1	1	0.5	1	1	0
40	“Corona Viruses and Computer Viruses: It’s Time for a Cyber Health Check-Up”	2020	National Law Review/Hardimanet al./Report article [92]	1	1	1	1	1	0
41	“Phishing scams, spam spike as hackers use coronavirus to prey on remote workers, stressed IT systems”	2020	CNBC/Eric/News article [93]	1	1	0.5	1	1	0
42	“Hackers, APTs Exploiting COVID-19 with Phishing Attacks, Fraud Schemes”	2020	healthitsecurity.com/Jessica Davis /news article [94]	0.5	0.5	0.5	0.5	1	0
43	“Threat Intel $\vert $ Cyber Attacks Leveraging the COVID-19/Coronavirus Pandemic”	2020	Sentinel LABS/Jim Walter/Report article [95]	1	1	1	0.5	1	0
44	“COVID-19’s Impact on Cybersecurity”	2020	Deloitte/Tope Aladenusi/Whitepaper [96]	1	1	0.5	0.5	1	0
45	“Global Landscape on Covid-19 Cyberthreat”	2020	INTERPOL/Whitepaper [97]	1	1	0.5	1	1	0
46	“COVID-19: Cyber Threat Analysis”	2020	United Nations Office on Drugs & Crime/Boismenu et al./Whitepaper [98]	1	1	0.5	1	1	0
47	“The COVID-19 Hackers Mind-set”	2020	ECHO/Whitepaper [99]	1	1	1	1	1	0
48	“Covid-19 Cyber Security Threats To MSMES”	2020	International chamber of commerce/Whitepaper [100]	1	1	0.5	0.5	1	1
49	“McAfee Labs COVID-19 Threats Report”	2020	McAfee/Report article [101]	1	1	0.5	1	1	0
50	“CYBERCRIME Threats during the COVID-19 pandemic”	2020	Global Initiative/Prem Mahadevan/Whitepaper [102]	1	1	0.5	1	1	0
51	“Catching the virus cybercrime, disinformation and the COVID-19 pandemic”	2020	Europol/Catherine de Bolle/Whitepaper [103]	1	1	0.5	0.5	1	0
52	“COVID-19: Impact on the Cyber Security Threat Landscape”	2020	Mouton and Coning/Whitepaper [104]	1	1	0.5	0.5	1	0

## References

[1] F. Mouton, L. Leenen, and H. S. Venter, “Social engineering attack examples, templates and scenarios,” Comput. Secur., vol. 59, pp. 186–209, Jun. 2016.

[2] K. Krombholz, H. Hobel, M. Huber, and E. Weippl, “Advanced social engineering attacks,” J. Inf. Secur. Appl., vol. 22, pp. 113–122, Jun. 2015, doi: 10.1016/j.jisa.2014.09.005.

[3] L. Xiangyu, L. Qiuyang, and S. Chandel, “Social engineering and insider threats,” in Proc. Int. Conf. Cyber-Enabled Distrib. Comput. Knowl. Discovery (CyberC), Oct. 2017, pp. 25–34.

[4] S. Abraham and I. Chengalur-Smith, “An overview of social engineering malware: Trends, tactics, and implications,” Technol. Soc., vol. 32, no. 3, pp. 183–196, Aug. 2010.

[5] D. Irani, M. Balduzzi, D. Balzarotti, E. Kirda, and C. Pu, “Reverse social engineering attacks in online social networks,” in Proc. Int. Conf. Detection Intrusions Malware, Vulnerability Assessment, 2011, pp. 55–74.

[6] Digital Guardian. (Jul. 6, 2015). What is Social Engineering? Defining and Avoiding Common Social Engineering Threats. Accessed: Sep. 2, 2020. [Online]. Available: https://digitalguardian.com/blog/what-social-engineering-defining-and-avoiding-common-social-engineering- threats

[7] 2019 Cyberthreat Defense Report |CyberEdge Group. Accessed: Sep. 3, 2020. [Online]. Available: https://cyber-edge.com/portfolio/2019-cyberthreat-defense-report/

[8] Proofpoint. (Apr. 6, 2018). The Human Factor 2019 Report—Modern Cyber Attacks. Accessed: Sep. 3, 2020. [Online]. Available: https://www.proofpoint.com/us/resources/threat-reports/human-factor

[9] Wikipedia. (Sep. 7, 2020). COVID-19 Pandemic. Accessed: Sep. 7, 2020. [Online]. Available: https://en.wikipedia.org/w/index.php?

[10] Coronavirus Disease (COVID-19)—World Health Organization. Accessed: Sep. 7, 2020. [Online]. Available: https://www.who.int/emergencies/diseases/novel-coronavirus-2019

[11] White Paper: Science and Technology Play a Vital Role in COVID-19 Combat. Accessed: Sep. 7, 2020. [Online]. Available: https://news.cgtn.com/news/2020-06-07/White-paper-Science-technology-play-a-vital-role-in-COVID-19-combat-R7C0geBkVG/index.html

[12] Learning Center. What is Social Engineering |Attack Techniques & Prevention Methods |Imperva. Accessed: Sep. 7, 2020. [Online]. Available: https://www.imperva.com/learn/application-security/social-engineering-attack/

[13] RLCS. (Apr. 24, 2018). Hacking Small Business and Organizations. Accessed: Sep. 7, 2020. [Online]. Available: https://redlion.co.ke/art-of-the-hack-hacking-small-business-and-organizations-2/

[14] A. Koyun and E. A. Janabi, “Social engineering attacks,” J. Multidisciplinary Eng. Sci. Technol., vol. 4, no. 6, p. 6, 2017.

[15] Social Engineering Attacks: An Augmentation of the Socio-Technical Systems Framework—ProQuest. Accessed: Sep. 10, 2020. [Online]. Available: https://search.proquest.com/docview/1781336033?pq-origsite=gscholar&fromopenview=true

[16] Security Through Education. Information Gathering. Accessed: Sep. 8, 2020. [Online]. Available: https://www.social-engineer.org/framework/information-gathering/

[17] H. Omotunde and R. Ibrahim, “A review of threat modelling and its hybrid approaches to software security testing,” ARPN J. Eng. Appl. Sci., vol. 10, no. 23, p. 17, 2015.

[18] A. Cullen and L. Armitage, “A human vulnerability assessment methodology,” in Proc. Int. Conf. Cyber Situational Awareness, Data Anal. Assessment (Cyber SA), Jun. 2018, pp. 1–2, doi: 10.1109/CyberSA.2018.8551371.

[19] Post Exploitation—An overview |ScienceDirect Topics. Accessed: Sep. 9, 2020. [Online]. Available: https://www.sciencedirect.com/topics/computer-science/post-exploitation

[20] Security Through Education. Typical Goals. Accessed: Sep. 10, 2020. [Online]. Available: https://www.social-engineer.org/framework/general-discussion/typical-goals/

[21] V. Garousi, M. Felderer, and T. Hacaloǧlu, “Software test maturity assessment and test process improvement: A multivocal literature review,” Inf. Softw. Technol., vol. 85, pp. 16–42, 5 2017.

[22] V. Garousi, M. Felderer, and M. V. Mäntylä, “Guidelines for including grey literature and conducting multivocal literature reviews in software engineering,” Inf. Softw. Technol., vol. 106, pp. 101–121, Feb. 2019.

[23] V. Garousi and M. V. Mäntylä, “When and what to automate in software testing? A multi-vocal literature review,” Inf. Softw. Technol., vol. 76, pp. 92–117, Aug. 2016, doi: 10.1016/j.infsof.2016.04.015.

[24] B. Kitchenham, R. Pretorius, D. Budgen, O. P. Brereton, M. Turner, M. Niazi, and S. Linkman, “Systematic literature reviews in software engineering—A tertiary study,” Inf. Softw. Technol., vol. 52, no. 8, pp. 792–805, 2010.

[25] C. Islam, M. A. Babar, and S. Nepal, “A multi-vocal review of security orchestration,” ACM Comput. Surv., vol. 52, no. 2, pp. 1–45, 5 2019, doi: 10.1145/3305268.

[26] Cyber Resilient Business |Accenture. Accessed: Oct. 11, 2020. [Online]. Available: https://www.accenture.com/ca-en/insights/cyber-security-index

[27] B. News. (Jun. 27, 2020). California University Paid $1.14 Million After Ransomware Attack—BNN Bloomberg. Accessed: Oct. 11, 2020. [Online]. Available: https://www.bnnbloomberg.ca/california-university-paid-1-14-million-after-ransomware-attack-1.1457176

[28] P. Muncaster. (Jun. 22, 2020). Online Fraudsters Steal 17m Over #COVID19 Lockdown. Infosecurity Magazine. Accessed: Oct. 11, 2020. [Online]. Available: https://www.infosecurity-magazine.com:443/news/online-fraudsters-steal-17-m/

[29] R. Nunes-Vaz, “Visualising the doubling time of COVID-19 allows comparison of the success of containment measures,” Global Biosecur., vol. 1, no. 3, Mar. 2020, Art. no. 3.

[30] J. Wiggen, “The impact of COVID-19 on cyber-crime and state-sponsored cyber activities,” Konrad-Adenauer-Stiftung, Bonn, Germany, Tech. Rep., 2020.

[31] Worldwide Cybersecurity Market to 2025—Impact of COVID-19 on the Industry—Research and Markets, Businesswire, San Francisco, CA, USA, Jun. 2020.

[32] Senetas. (Mar. 20, 2020). How COVID-19 Threatens IT Security: Cyber-Threats to Remote Working. Accessed: Oct. 12, 2020. [Online]. Available: https://www.senetas.com/how-covid-19-threatens-it-security-cyber-threats-to-remote-working/

[33] Remote Working During Coronavirus Pandemic Leads to Rise in Cyber-Attacks, Say Security Professionals, Portwigger, The Daily Swig |Cybersecurity News Views, Jul. 2020.

[34] TechRepublic. Br, on V. in S. on April 21, 2020, and 11:25 Am Pst, COVID-19 Lockdowns are Causing a Huge Spike in Data Breaches. Accessed: Oct. 12, 2020. [Online]. Available: https://www.techrepublic.com/article/covid-19-lockdowns-are-causing-a-huge-spike-in-data-breaches/

[35] IAITAM—International Association of Information Technology Managers Official Home Page. Accessed: Oct. 12, 2020. [Online]. Available: https://iaitam.org/

[36] D. Palmer. Thousands of COVID-19 Scam and Malware Sites are Being Created on a Daily Basis. ZDNet. Accessed: Oct. 12, 2020. [Online]. Available: https://www.zdnet.com/article/thousands-of-covid-19-scam-and-malware-sites-are-being-created-on-a-daily-basis/

[37] J. W. A. Markowitz. Coronavirus Scams—Beware Fake Claims, Phony Websites. AARP. Accessed: Oct. 12, 2020. [Online]. Available: http://www.aarp.org/money/scams-fraud/info-2020/coronavirus.html

[38] Beware of These Coronavirus Scams. Accessed: Oct. 12, 2020. [Online]. Available: https://us.norton.com/internetsecurity-online-scams-coronavirus-phishing-scams.html

[39] Ready-Made COVID-19 Themed Phishing Templates Copy Government Websites Worldwide |Proofpoint US, Proofpoint, Sunnyvale, CA, USA, 2020.

[40] Unit42. (Apr. 22, 2020). Studying How Cybercriminals Prey on the COVID-19 Pandemic. Accessed: Oct. 12, 2020. [Online]. Available: https://unit42.paloaltonetworks.com/how-cybercriminals-prey-on-the-covid-19-pandemic/

[41] Osler, Hoskin & Harcourt LLP. Privacy and Security Challenges in the Wake of COVID-19. Accessed: Oct. 12, 2020. [Online]. Available: http://www.osler.com/en/resources/critical-situations/2020/privacy-and-security-challenges-in-the-wake-of-covid-19

[42] S. Schinagl, “What do we know about information security governance? ‘From the basement to the boardroom’: Towards digital security governance,” Res. IT-Auditing Multidisciplinary View Ed., p. 135, Feb. 2020.

[43] Information Age. (Sep. 25, 2020). From Adoption to Understanding: AI in Cyber Security Beyond COVID-19. Accessed: Oct. 14, 2020. [Online]. Available: https://www.information-age.com/from-adoption-understanding-ai-cyber-security-beyond-covid-19-123491785/

[44] Smoke and Mirrors: Do AI and Machine Learning Make a Difference in Cybersecurity? Accessed: Oct. 14, 2020. [Online]. Available: https://mypage.webroot.com/ai-ml-survey-report-2020.html

[45] (Aug. 31, 2020). How COVID-19 Will Help Lead the Shift to AI-First Cybersecurity, IoT For All. Accessed: Oct. 14, 2020. [Online]. Available: https://www.iotforall.com/how-covid-19-will-help-lead-the-shift-to-ai-first-cybersecurity

[46] L. Chan, I. Morgan, H. Simon, F. Alshabanat, D. Ober, J. Gentry, D. Min, and R. Cao, “Survey of AI in cybersecurity for information technology management,” in Proc. IEEE Technol. Eng. Manage. Conf. (TEMSCON), Jun. 2019, pp. 1–8, doi: 10.1109/TEMSCON.2019.8813605.

[47] Q. Pham, D. C. Nguyen, T. Huynh-The, W. Hwang and P. N. Pathirana, “Artificial intelligence (AI) and big data for coronavirus (COVID-19) pandemic: A survey on the state-of-the-arts,” IEEE Access, vol. 8, pp. 130820–130839, 2020, doi: 10.1109/ACCESS.2020.3009328. PMC8545324 34812339

[48] N. Janoschek. (Nov. 28, 2016). Big Data Security Analytics: A Weapon Against Cyber Security Attacks? [Video]. BI Survey. Accessed: Oct. 15, 2020. [Online]. Available: http://bi-survey.com/big-data-security-analytics

[49] Network Security Needs Big Data. Accessed: Oct. 15, 2020. [Online]. Available: https://ahmedbanafa.blogspot.com/2014/10/network-security-needs-big-data.html

[50] L. Wang and R. Jones, “Big data analytics in cyber security: Network traffic and attacks,” J. Comput. Inf. Syst., pp. 1–8, 2020.

[51] S. Acharath, “Cybersecurity and data analytics-self reflection,” Medium, Towards Datacsi., Jun. 2020.

[52] Securing the Internet of Things (IoT) With Blockchain. Accessed: Oct. 14, 2020. [Online]. Available: https://ahmedbanafa.blogspot.com/2016/08/securing-internet-of-things-iot-with.html

[53] Second Line of Defense for Cybersecurity: Blockchain. Accessed: Oct. 14, 2020. [Online]. Available: https://ahmedbanafa.blogspot.com/2018/02/second-line-of-defense-for.html

[54] R. Naidoo, “A multi-level influence model of COVID-19 themed cybercrime,” Eur. J. Inf. Syst., vol. 29, no. 3, pp. 306–321, 2020, doi: 10.1080/0960085X.2020.1771222.

[55] A. Alzahrani, “Coronavirus social engineering attacks: Issues and recommendations,” Int. J. Adv. Comput. Sci. Appl., vol. 11, no. 5, pp. 154–161, 2020, doi: 10.14569/IJACSA.2020.0110523.

[56] P. Chapman, “Are your IT staff ready for the pandemic-driven insider threat?” Netw. Secur., vol. 2020, no. 4, pp. 8–11, Apr. 2020.

[57] S. Hakak, W. Z. Khan, M. Imran, K.-K.-R. Choo, and M. Shoaib, “Have you been a victim of COVID-19-related cyber incidents? Survey, taxonomy, and mitigation strategies,” IEEE Access, vol. 8, pp. 124134–124144, 2020. 34192113 10.1109/ACCESS.2020.3006172 PMC8043498

[58] R. He, H. Wang, P. Xia, L. Wang, Y. Li, L. Wu, Y. Zhou, X. Luo, Y. Guo, and G. Xu, “Beyond the virus: A first look at coronavirus-themed mobile malware,” 2020, arXiv:2005.14619. [Online]. Available: http://arxiv.org/abs/2005.14619 10.1007/s10664-021-09974-4 PMC8196937 34149303

[59] H. Singh Lallie, L. A. Shepherd, J. R. C. Nurse, A. Erola, G. Epiphaniou, C. Maple, and X. Bellekens, “Cyber security in the age of COVID-19: A timeline and analysis of cyber-crime and cyber-attacks during the pandemic,” 2020, arXiv:2006.11929. [Online]. Available: http://arxiv.org/abs/2006.11929 10.1016/j.cose.2021.102248 PMC9755115 36540648

[60] P. Xia, H. Wang, X. Luo, L. Wu, Y. Zhou, G. Bai, G. Xu, G. Huang, and X. Liu, “Don’t fish in troubled waters! Characterizing coronavirus-themed cryptocurrency scams,” 2020, arXiv:2007.13639. [Online]. Available: http://arxiv.org/abs/2007.13639

[61] T. Weil and S. Murugesan, “IT risk and resilience—Cybersecurity response to COVID-19,” IT Prof., vol. 22, no. 3, pp. 4–10, 5 2020.

[62] K. Okereafor and O. Adelaiye, “Randomized cyber attack simulation model: A cybersecurity mitigation proposal for post COVID-19 digital era,” Int. J. Recent Eng. Res. Develop., vol. 5, no. 7, pp. 61–72, Jul. 2020.

[63] C. Tran, “Recommendations for ordinary users from mitigating phishing and cybercrime risks during COVID-19 pandemic,” Jun. 2020, arXiv: 2006.11929v1. [Online]. Available: https://arxiv.org/abs/2006.11929

[64] K. Okereafor and O. Adebola, “Tackling the cybersecurity impacts of the coronavirus outbreak as a challenge to Internet safety. Figshare,” J. Contribution, 2020, doi: 10.6084/m9.figshare.12479159.v1.

[65] N. A. Khan, S. N. Brohi, and N. Zaman, “Ten deadly cyber security threats amid COVID-19 pandemic,” TechRxiv, to be published, doi: 10.36227/techrxiv.12278792.v1.

[66] Situational Awareness: Cyber Threats Heightened by COVID-19 and How to Protect Against Them. Accessed: Oct. 17, 2020. [Online]. Available: https://www.google.com/search?

[67] Developing Story: COVID-19 Used in Malicious Campaigns Security News—Trend Micro, Nov. 2020.

[68] JD Supra. Flattening the Scam Curve: Be Prepared for Uptick in COVID-19 Social Engineering Cyber Attacks. Accessed: Oct. 17, 2020. [Online]. Available: https://www.jdsupra.com/legalnews/flattening-the-scam-curve-be-prepared-74767/

[69] Criminals Exploit COVID-19 Fears to Launch ’Unprecedented Wave’ of Global Cyberattacks |Arab News. Accessed: Oct. 17, 2020. [Online]. Available: https://www.arabnews.com/node/1648851/saudi-arabia

[70] COVID-19 Exploited by Malicious Cyber Actors |CISA. Accessed: Oct. 17, 2020. [Online]. Available: https://us-cert.cisa.gov/ncas/alerts/aa20-099a

[71] PricewaterhouseCoopers. How to Protect Your Companies From Rising Cyber Attacks and Fraud Amid the COVID-19 Outbreak. Accessed: Oct. 17, 2020. [Online]. Available: https://www.pwc.com/us/en/library/covid-19/cyber-attacks.html

[72] Panda Security Mediacenter. 43 COVID-19 Cybersecurity Statistics. Accessed: Oct. 17, 2020. [Online]. Available: https://www.pandasecurity.com/mediacenter/news/covid-cybersecurity-statistics/

[73] Exploiting a Crisis: How Cybercriminals Behaved During the Outbreak, Microsoft Secur., Redmond, WA, USA, Jun. 2020.

[74] Cybercriminals are Leveraging Coronavirus to Boost Profit, L. LLC, LIFARS, New York, NY, USA, Mar. 2020.

[75] Verisk. Cyber Security in Uncertain Times. Accessed: Oct. 17, 2020. [Online]. Available: https://www.verisk.com/resources/COVID-19/cyber-security-in-uncertain-times/

[76] Ransomware Groups Continue to Target Healthcare, Critical Services; Here’s How to Reduce Risk, Microsoft Secur., Redmond, WA, USA, Apr. 2020.

[77] Coronavirus (COVID-19) Cyber Crimes & Threats |LookingGlass, LookingGlass Cyber Solutions Inc., Reston, VA, USA, Mar. 2020.

[78] LORCA. (5 6, 2020). Social Engineering Attacks and COVID-19. Accessed: Oct. 17, 2020. [Online]. Available: https://www.lorca.co.uk/social-engineering-attacks-and-covid-19/

[79] Why the Coronavirus Pandemic Presents a Golden Opportunity for Hackers, Secur. Info Watch, Mountain View, CA, USA, Mar. 2020.

[80] Bad Actors Have Adapted Well to the Pandemic Crisis. Accessed: Oct. 17, 2020. [Online]. Available: https://www.govtech.com/security/Bad-Actors-Have-Adapted-Well-to-the-Pandemic-Crisis.html

[81] F-Secure Blog. (Mar. 30, 2020). Latest COVID-19-Related Cyber Security News: Hospitals Under Attack. Accessed: Oct. 17, 2020. [Online]. Available: https://blog.f-secure.com/covid-19-cyber-security/

[82] COVID-19 Social Engineering Attacks |CSO Online. Accessed: Oct. 17, 2020. [Online]. Available: https://www.csoonline.com/article/3533339/covid-19-social-engineering-attacks.html

[83] BioCatch. Types of Social Engineering Attacks [Recent 2020 Scams]. Accessed: Oct. 11, 2020. [Online]. Available: https://www.biocatch.com/blog/types-social-engineering-attacks

[84] Coronavirus: Its Four Most Prevalent Cyber Threats—Security Boulevard. Accessed: Oct. 11, 2020. [Online]. Available: https://securityboulevard.com/2020/03/coronavirus-its-four-most-prevalent-cyber-threats/

[85] SDxCentral. (Mar. 27, 2020). Security Experts Battle Hackers, COVID-19 Cyberattacks. Accessed: Oct. 17, 2020. [Online]. Available: https://www.sdxcentral.com/articles/news/security-experts-battle-hackers-covid-19-cyberattacks/2020/03/

[86] Coronavirus: Cyber Attacks on Banks Seen Spiking, Says Carbon Black. Accessed: Oct. 17, 2020. [Online]. Available: https://www.computerweekly.com/news/252481684/Coronavirus-Cyber-attacks-on-banks-seen-spiking-says-Carbon-Black

[87] S. Williams. Trend Micro: COVID-19 Related Malware and Spam on the Rise. Accessed: Oct. 17, 2020. [Online]. Available: https://securitybrief.eu/story/trend-micro-covid-19-related-malware-and-spam-on-the-rise

[88] Ransomware Gangs and COVID-19 Cyberattacks Dominate the Threat Landscape. Accessed: Oct. 17, 2020. [Online]. Available: https://intsights.com/blog/ransomware-gangs-and-covid-19-cyberattacks-dominate-the-threat-landscape

[89] Techerati. Cyber Attacks in the Pandemic era: More of the Same? Accessed: Oct. 17, 2020. [Online]. Available: https://techerati.com/features-hub/opinions/cyber-attacks-in-the-pandemic-era-more- of-the-same/

[90] COVID-19 Cybersecurity Alerts |Coronavirus Phishing Scam |Covid 19 Malware |Cyware |Blog, Cyware Labs, Bengaluru, India, Aug. 2020.

[91] Cybersecurity’s Dual Mission During the Coronavirus Crisis—Google Search. Accessed: Oct. 17, 2020. [Online]. Available: https://www.google.com/search?

[92] The National Law Review. Corona Viruses and Computer Viruses: It’s Time for a Cyber Health Check-Up. Accessed: Oct. 17, 2020. [Online]. Available: https://www.natlawreview.com/article/corona-viruses-and-computer-viruses-it-s-time-cyber-health-check

[93] E. Rosenbaum. (Mar. 20, 2020). Phishing Scams, Spam Spike as Hackers Use Coronavirus to Prey on Remote Workers, Stressed IT Systems. CNBC. Accessed: Oct. 17, 2020. [Online]. Available: https://www.cnbc.com/2020/03/20/phishing-spam-spike-as-hackers-use-coronavirus-to-hit-remote-work.html

[94] HealthITSecurity. (Apr. 9, 2020). Hackers, APTs Exploiting COVID-19 With Phishing Attacks, Fraud Schemes. Accessed: Oct. 17, 2020. [Online]. Available: https://healthitsecurity.com/news/hackers-apts-exploiting-covid-19-with-phishing-attacks-fraud-schemes

[95] SentinelLabs. (Sep. 4, 2020). Threat Intel |Cyber Attacks Leveraging the COVID-19/CoronaVirus Pandemic. Accessed: Oct. 17, 2020. [Online]. Available: https://labs.sentinelone.com/threat-intel-update-cyber-attacks-leveraging-the-covid-19-coronavirus-pandemic/

[96] Deloitte Nigeria. COVID-19’s Impact on Cybersecurity. Accessed: Oct. 17, 2020. [Online]. Available: https://www2.deloitte.com/ng/en/pages/risk/articles/covid-19-impact-cybersecurity.html

[97] COVID-19 Cyberthreats. Accessed: Oct. 17, 2020. [Online]. Available: https://www.interpol.int/en/Crimes/Cybercrime/COVID-19-cyberthreats

[98] COVID-19: Cyber Threat Analysis—Google Search. Accessed: Oct. 17, 2020. [Online]. Available: https://www.google.com/search?

[99] The COVID-19 Hackers Mind-set—Google Search. Accessed: Oct. 17, 2020. [Online]. Available: https://www.google.com/search?

[100] ICC—International Chamber of Commerce. COVID-19: Cyber Security Threats to MSMEs. Accessed: Oct. 17, 2020. [Online]. Available: https://iccwbo.org/publication/covid-19-cyber-security-threats-to-msmes/

[101] McAfee Labs COVID-19 Threats Report—Google Search. Accessed: Oct. 17, 2020. [Online]. Available: https://www.google.com/search?

[102] Global Initiative. Cybercrime: Threats During the Covid-19 Pandemic. Accessed: Oct. 17, 2020. [Online]. Available: https://globalinitiative.net/analysis/cybercrime-covid-19/

[103] Europol. Catching the Virus Cybercrime, Disinformation and the COVID-19 Pandemic. Accessed: Oct. 17, 2020. [Online]. Available: https://www.europol.europa.eu/publications-documents/catching-virus-cybercrime-disinformation-and-covid-19-pandemic

[104] F. Mouton and A. De Coning, “COVID-19: Impact on the cyber security threat landscape,” Tech. Rep., Mar. 2020.

[105] M. Perc, N. G. Miksic, M. Slavinec, and A. Stozer, “Forecasting COVID-19,” Frontiers Phys., vol. 8, p. 127, Apr. 2020.

[106] M.-G. Hâncean, M. Perc, and J. Lerner, “Early spread of COVID-19 in Romania: Imported cases from Italy and human-to-human transmission networks,” Roy. Soc. Open Sci., vol. 7, no. 7, Jul. 2020, Art. no. 200780. 10.1098/rsos.200780 PMC7428275 32874663

[107] S. Momtazmanesh, “All together to fight COVID-19,” Amer. J. Tropical Med. Hygiene, vol. 102, no. 6, pp. 1181–1183, Jun. 2020, doi: 10.4269/ajtmh.20-0281. PMC7253116 32323644

